# Australian public health COVID-19 messaging is missing its mark in some vulnerable communities and people who reject COVID-19 safety advice

**DOI:** 10.7189/jogh.12.05037

**Published:** 2022-09-03

**Authors:** Megan Jepson, Glen A Whittaker, Lauren Robins, Katrina M Long, Cylie M Williams, Grant Russell, Keith D Hill, Libby Callaway, Jim Hlavac, Louisa Willoughby, Terry P Haines

**Affiliations:** 1 School of Primary and Allied Health Care, Faculty of Medicine, Nursing, and Health Sciences, Monash University, Frankston, Victoria, Australia; 2 Discipline of Podiatry, School of Allied Health, Human Services and Sport, La Trobe University, Melbourne, Victoria, Australia; 3 Peninsula Health, Allied Health, Victoria, Australia; 4 Department of General Practice, Faculty of Medicine Nursing and Health Sciences, Monash University, Victoria, Australia; 5 Rehabilitation Ageing and Independent Living (RAIL) Research Centre, Faculty of Medicine, Nursing, and Health Sciences, Monash University, Victoria, Australia; 6 Occupational Therapy Department, Faculty of Medicine, Nursing and Health Sciences, Monash University, Frankston, Victoria; 7 Translation and Interpreting Studies, Faculty of Arts, Monash University, Victoria, Australia; 8 Linguistics, Faculty of Arts, Monash University, Melbourne, Victoria, Australia

## Abstract

**Background:**

There are groups in our community who may be more vulnerable to contracting, transmitting, or experiencing negative health impacts of COVID-19 than the general community. They may also have greater difficulty accessing, accepting, and acting upon COVID-19 public health information. Our aim was to understand if vulnerable communities and those who express “COVID-risk” behavioural intentions seek and respond differently to COVID-19 public health information.

**Methods:**

This observational, cross-sectional study recruited adults aged over 18 years from the Australian general community and six community groups (people with disabilities and their caregivers, Aboriginal and Torres Strait Islanders, aged care workers, street-based sex workers, refugees and asylum seekers, and the deaf and hard of hearing). We investigated attitudes and beliefs about COVID-19 public health messages. We identified factors associated with the respondent’s perception of the ease of finding information and understanding it, and its relevance to them. We also examined latent classes that were developed based on attitudes to public health measures and vulnerable group categories, along with demographic variables.

**Results:**

We received 1444 responses (n = 1121 general community; n ≥50 for each vulnerable group). The vulnerable groups examined found COVID-19 public health messages as easy, if not easier, to find and understand than the general community. Four latent classes were identified: COVID-safe mask wearers (10% of sample), COVID-safe test takers (56%), COVID-risk isolators (19%) and COVID-risk visitors (15%). The COVID-risk classes (34% of sample) were less likely to consider COVID-19 information easy to find, understandable, and relevant.

**Conclusions:**

Additional public health messaging strategies may be needed for targeting people with “COVID-risk” beliefs and attitudes who appear across the community (general and vulnerable groups) rather than just targeting specific cultural or other groupings that we think may be vulnerable. COVID-risk classes identified through this study were not defined by demographic characteristics or cultural groupings, but were spread across vulnerable communities and the general community. Different approaches for tailoring and delivery of specific public health information for these groups are needed.

COVID-19 is likely the worst public health crisis in Australia since the 1919 Spanish Flu [1]. Effective management of the COVID-19 pandemic and future pandemics in Australia requires significant and sustained population-wide behaviour change. There is evidence that mass media interventions delivering public health information can influence behaviours such as treatment-seeking, behavioural intentions, knowledge, and awareness [2,3]. It is plausible that well-informed, mass-media information and communication campaigns delivering public health information could be a useful tool for combatting the COVID-19 pandemic.

The delivery of COVID-19 public health information using a “one size fits all” approach may not be effective for everyone in the community, particularly people from communities who are vulnerable to contracting COVID-19. The Australian government has provided targeted resources for some vulnerable communities, either directly or via funding to peak bodies [4-11]. However, the provision of this information is somewhat fragmented, with the content and communication styles varying between states. It is also unclear to what extent the resources have been tailored to the cultural needs of each community, as many appear to be direct translations of English-language resources without cultural adaptation. Previous research has identified that specific groups with differing abilities, needs, languages, and/or cultural leanings across issues such as individualism and collectivism respond differently to public health information [12,13]. For example, recent Australian data indicate the vulnerability of some communities to COVID-19, with people who have significant disability and/or underlying health conditions, and those from culturally and linguistically diverse backgrounds disproportionally impacted [14]. In addition, people with specific socio-economic backgrounds and/or occupational choices are at greater risk [15,16], including where an occupation may lead to expression of “COVID-risk” behavioural intentions [17]. **Table 1** summarises reasons why certain community groups are vulnerable to contracting COVID-19.

**Table 1:** Vulnerable community groups included in the study and the reasons for vulnerability

COVID-19 risk factors & communication challenges	Community subgroup					
**Aboriginal & Torres Strait Islander people**	**People living with disability & their carers**	**Refugee & asylum seekers**	**Deaf, hard of hearing people**	**Aged care workers**	**Street-based sex workers**
Higher exposure risk		**+** due to personal care tasks [3,18]			**+** work environment	**+** work environment [19,20]
Higher rates of health conditions & need for health services	**+** [3,18]	**+** [3,18]	**+** [21]			**+** [19]
Difficulty accessing health care	**+** [3]		**+** [21]			**+** [19,20]
Higher rates of limited English proficiency			**+**	**+**	**+**	
Lower rates of digital literacy and access to the Internet or Internet-enabled devices		**+** [22,23]				
Limited accessible COVID-19 information		**+** [18]		**+** [24]		
Loss of educational support and access		**+**	**+**	**+** [25]		
Different cultural understandings of illness	**+**		**+**			
Lower socio-economic status	**+** [26]	**+** [18]	**+** [21]			**+** [19]
Higher rates of contract, casual or precarious work		**+** [27]		**+** [28,29]	**+**	**+** [19,20]
Housing situation	**+** Larger, multi-family households [26]		**+** Larger, multi-family households			**+** Lack of secure housing [20]

Vulnerable subgroup-specific approaches, such as the ‘For all of us’ campaign that targeted Aboriginal and Torres Strait Islander peoples in Australia [30], may be a better approach to public-health messaging than the one-size-fits-all mass media approach. However, it would be presumptive to assume that all people within these community sub-groupings receive and respond the same way to public health information. Instead, it is plausible that “latent groups” exist that cut across vulnerable communities and the general community, who are characterised by a shared tendency toward behaviours and attitudes that may negatively impact or be unhelpful in reducing the spread and impact of COVID-19. If these latent subgroupings exist, they may potentially be described and targeted with tailored messaging which may be more effective than approaches focused on cultural, ethnic, or other known groups.

This study aimed to: i) identify and characterise latent classes who report behavioural intentions not conducive to minimising the risk of spreading COVID-19, and ii) understand how the latent classes, the general community, and vulnerable communities respond to COVID-19 public health information.

## METHODS

### Design

An observational, cross-sectional, online survey with quota sampling was used to collect data. The design of the study and the customised survey were informed by a steering committee. This study report is presented in accordance with the Strengthening the Reporting of Observational Studies in Epidemiology (STROBE) checklist [31]. Ethical approval was granted by the Human Research Ethics Committee at Monash University (HREC 24040) and each participant provided informed consent through a check box at the beginning of the survey.

We used quota sampling to ensure a minimum sample size of 50 participants across six community subgroups. These groups were considered particularly vulnerable to contracting, transmitting, or experiencing particularly poor health outcomes from COVID-19, making them a high priority for public health interventions. They were Aboriginal and Torres Strait Islander peoples, people living with a disability and their carers, refugee and asylum seekers, deaf/hard of hearing people, aged care workers, and street-based sex workers. A full justification for selection of each is presented in **Table 1**. We also recruited a sample from the general community where no quota was set.

### Patient and public involvement

A steering committee was created prior to data collection that comprised Monash University researchers with experience working with at least one of the identified vulnerable communities as well as community representatives including CEO’s and senior managers and peer workers of organisations associated with the vulnerable communities. These vulnerable communities and associated organisations included: Able Australia and Wallara and Yooralla (people living with disability and their carers); Donwood Community Aged Care Services (aged care workers); enliven (refugee and asylum seekers); Expression Australia and Able Australia (deaf and hard of hearing people); Peninsula Health (Aboriginal and Torres Strait Islander people); and St Kilda Gatehouse (street-based sex workers). The Australian Government Department of Health was represented on the steering committee by the Co-Assistant Secretary, Public Information Branch. The representatives of the organisational sub-groups from the steering committee assisted with recruitment.

### Participants and setting

Participants were eligible to participate in our survey if they were aged over 18 years, resided in Australia and were a member of the general community or one of our vulnerable community groups. In Australia, COVID-19 public health information has primarily been delivered through daily press conferences by State Premiers and Chief Medical Officers. These press conferences have often been broadcast live on public television and radio stations with summaries presented in nightly news bulletins, websites and social media platforms. Public service announcement commercials from the Australian Government Department of Health have also been employed (eg, the ‘Arm yourself against COVID-19’ campaign [32] and ‘Don’t be complacent’ campaign [33], with sub-group specific approaches also used [30].

### Procedure

Participants from the vulnerable community groups were recruited via email, social media, and in-person with the assistance of members of partner organisations represented on our steering committee. Specific scripts were developed to inform participants about the study and obtain informed consent. Scripts were tailored to each community group (eg, translations to different languages for refugee and asylum seekers and translation to Auslan for the deaf and hard-of-hearing). Participants who could not complete the survey in the online format were offered an interview or paper-based survey. Participants of the vulnerable communities were offered an honorarium of an AU$50 gift card for their time, and the broader community entered a prize draw to win one of 20 AU$50 gift cards. Specific details of the recruitment methods for each vulnerable community are outlined in Table S1 in the **Online Supplementary Document**. Online advertising was used to recruit participants from the general community.

### Outcome measures

Data were anonymously collected via a customised survey using either Qualtrics® software (Qualtrics, Provo, UT, USA) or its paper-based format with extracted questions. The specific items used to collect data for this study are included in Appendix S2 in the **Online Supplementary Document**. Questions were developed to collect demographic information (eg, age, gender, state, employment categories), sources of exposure to COVID-19 information (eg, newspapers, radio), opinions regarding trust in people/groups (eg, general practitioners, politicians), COVID-safe behaviours (eg, likelihood to get tested if you develop a fever), and if COVID-19 information in Australia was “easy to find”, “easy to understand”, and “relevant to you”. Likert-style scaling of item responses (4 or 5-point) was used to capture participant responses.

### Statistical analysis

Survey data were imported into StataSE version 15.0 (StataCorp LLC, Texas, United States of America) to perform statistical analyses. Sample characteristics were reported descriptively.

To identify “latent sub-groups”, we used a four-step latent class analysis approach [34]. In step one, we selected survey items where participants reported behavioural intentions towards several “COVID-safe” behaviours which we used to build the latent class model. Survey items that represented important COVID-safe behaviours were selected by the steering group to have the lowest proportion of extreme responses (to avoid ceiling effects) and to minimise collinearity (to minimise redundancy within the model). These items examined the likelihood of 1) mask wearing outdoors when a person could not socially distance, 2) spending time with others that the person does not live with if cough symptoms developed, 3) seeking testing if cough symptoms developed, 4) visiting public places if cough symptoms developed, and 5) checking-in to public venues using QR codes. Responses for each item were dichotomised (merging of “extremely likely” and “likely” response categories, and “neutral” to “extremely unlikely” categories) to minimise analytic complexity and increase ease of interpretation. In step two, latent class models were built with two, three, four, and then five classes and the model with the lowest value for the Akaike Information Criterion was selected. Latent class marginal means were calculated for each class, telling us what proportion of participants that fit in each class responded positively to each item. For step three, each participant was mapped to a class by creating dichotomous class membership dummy variables. Finally, in step four, differences between each class in demographic characteristics, exposure to COVID-19 information, and trust in COVID-19 information sources were described. Multiple logistic regression models were constructed using a backward elimination approach to identify these defining characteristics [35]. Variables were also removed where multicollinearity (variance inflation factor >8.0) was identified.

Responses from each latent class and each vulnerable population subgroup to the questions examining whether COVID-19 information in Australia was “easy to find”, “easy to understand”, and “relevant to you” were compared. Survey responses were compared between latent classes using pair-wise ordered logit regression analyses and vulnerable groups were compared to the general community using a single multivariable ordered logit regression.

Three multivariable, ordered logistic regression models were constructed to identify which latent classes and demographic variables (including vulnerable group membership) were independently associated with COVID-19 information being “easy to find”, “easy to understand”, and “relevant to you”. A backwards, stepwise variable selection approach was employed with all variables commencing in the model, then being removed (based on having the highest *P*-value) one at a time with the model then re-run until all variables remaining in the model had a *P*-value <0.05. Variables excluded from the model were then re-entered to check if their inclusion better optimised the Akaike Information Criterion and excluded if they did not.

## RESULTS

Survey data were collected between May 2021 and July 2021. A total of 1444 people consented to participate, and the characteristics of the participants are outlined in **Table 2**. Data were incomplete for 346 participants (24%). There was no uniform distribution across our age group and gender characteristics within several vulnerable subgroups or with our general community sample.

**Table 2:** Participant characteristics

		Community subgroups						
**Characteristic**	**All participants**	**Community (n = 1121)**	**People with a disability and their carers (n = 53)**	**Aged care workers (n = 53)**	**Refugee and asylum seekers (n = 54)**	**Deaf/hard of hearing (n = 61)**	**Aboriginal and Torres Strait Islander people (n = 50)**	**Street-based sex workers (n = 50)**
Age (years, n = 1368), n (%)*								
Less than 30 y	136 (9)	72 (6)	11 (21)	12 (22)	16 (30)	12 (20)	8 (16)	5 (10)
30-39	396 (28)	323 (29)	13 (25)	9 (16)	12 (22)	9 (15)	10 (20)	20 (40)
40-49	252 (18)	169 (15)	8 (15)	16 (29)	5 (9)	18 (30)	15 (30)	21 (42)
50-59	237 (17)	179 (16)	15 (28)	11 (20)	13 (24)	10 (16)	7 (14)	2 (4)
60-69	216 (15)	190 (17)	4 (8)	3 (5)	5 (9)	8 (13)	5 (10)	1 (2)
70-79	116 (8)	108 (10)	2 (4)	0 (0)	1 (2)	0 (0)	4 (8)	1 (2)
80 y or over	15 (1)	14 (1)	0 (0)	0 (0)	0 (0)	0 (0)	1 (2)	0 (0)
Gender (n = 1373), n (%)*								
Male	230 (16)	155 (14)	17 (32)	8 (15)	18 (33)	8 (13)	24 (48)	0 (0)
Female	1118 (81)	895 (80)	35 (66)	43 (78)	33 (61)	46 (75)	21 (42)	45 (90)
Non-binary	9 (0.7)	3 (0.27)	1 (2)	0 (0)	1 (2)	0 (0)	4 (8)	0 (0)
Prefer not to say	13 (0.9)	3 (0.27)	0 (0)	0 (0)	1 (2)	4 (7)	1 (2)	4 (8)
My gender is not listed	3 (0.2)	2 (0.18)	0 (0)	0 (0)	0 (0)	0 (0)	0 (0)	1 (2)
Australian born (n = 1376), n (%)*								
Yes	852 (61)	640 (57)	41 (77)	29 (53)	2 (4)	49 (80)	50 (100)†	41 (82)
Identify as religious (n = 1376), n (%)*								
Catholic	248 (18)	520 (46)	25 (47)	20 (36)	2 (4)	32 (53)	39 (78)	32 (64)
Other Christian	203 (14)	187 (17)	16 (30)	7 (13)	14 (26)	14 (23)	5 (10)	5 (10)
Islam	80 (5)	180 (16)	5 (9)	4 (7)	0 (0)	6 (10)	2 (4)	6 (12)
Other	175 (12)	52 (5)	0 (0)	6 (11)	22 (41)	0 (0)	0 (0)	0 (0)
Ability to read English (n = 1368), n (%)*								
Very well	1219 (89)	1037 (93)	33 (62)	42 (76)	20 (37)	19 (31)	32 (64)	36 (72)
Well	116 (8)	19 (2)	11 (21)	8 (15)	20 (37)	32 (52)	13 (26)	13 (26)
Not well	30 (2)	0 (0)	7 (13)	0 (0)	11 (20)	7 (11)	4 (8)	1 (2)
Not at all	3 (0.2)	0 (0)	2 (4)	0 (0)	0 (0)	0 (0)	1 (2)	0 (0)
**Exposure to COVID-19 information in the past month, median (IQR)‡**								
Television	3 (2, 4)	3 (2, 4)	3 (2, 4)	4 (3, 4)	3 (2, 4)	3 (2, 4)	3.5 (2, 4)	3 (2, 4)
Social media	3 (2, 4)	3 (2, 4)	2 (1, 3)	3 (2, 4)	3 (2, 4)	3 (2, 4)	2 (1, 4)	1 (1, 2)
Friends	3 (2, 3)	3 (2, 4)	3 (2, 4)	3 (3, 4)	3 (2, 4)	3 (2, 4)	3 (2, 3)	2 (1, 3)
Newspapers	2 (1, 3)	2 (1, 3)	1 (1, 3)	2 (1, 3)	1.5 (1, 2)	2 (1, 3)	1 (1, 2)	1 (1, 2)
Radio	2 (1, 3)	2 (1, 3)	2 (2, 4)	3 (2, 4)	2 (1, 3)	1 (1, 1)	3 (2, 3)	1.5 (1, 3)
Government	2 (1, 3)	2 (1, 3)	1 (1, 3)	3 (2, 4)	2 (2, 3)	2 (2, 3)	2 (1, 3)	1 (1, 2)
Workplaces	2 (1, 3)	2 (1, 3)	3 (1, 4)	4 (3, 4)	2 (1, 3)	3 (2, 3)	2 (1, 3)	1 (1, 1)
Community/religious gatherings	1 (1, 2)	1 (1, 1)	1 (1, 2)	2 (1, 2)	2 (1, 3)	1 (1, 2)	1 (1, 3)	1 (1, 1)
**Trust in COVID-19 information, median (IQR)‡**								
General practitioners	4 (3, 4)	4 (3, 4)	4 (3, 4)	4 (3, 4)	3 (2, 4)	3 (2, 4)	3 (2, 4)	2 (1, 3)
Scientists	4 (3, 4)	4 (3, 4)	3 (3, 4)	4 (3, 4)	3 (2, 4)	3 (2, 4)	3 (1, 4)	3 (1, 3)
Chief Medical Officers	3 (2, 4)	3 (2, 4)	3 (3, 4)	4 (3, 4)	4 (2, 4)	3 (2, 4)	2 (1, 3)	2 (1, 3)
Other health care professionals	3 (2, 4)	3 (3, 4)	3 (3, 4)	3 (3, 4)	3 (2, 4)	3 (2, 4)	3 (2, 3.5)	2 (1, 4)
Politicians	3 (2, 3)	3 (2, 3)	3 (2, 3)	3 (2, 4)	3 (2, 4)	3 (2, 3)	1 (1, 3)	2 (1, 2.5)
Friends	3 (2, 3)	3 (2, 3)	3 (2, 4)	3 (2, 3)	3 (2, 4)	3 (2, 4)	2 (2, 3)	3 (1, 3)
Family	3 (2, 3)	3 (2, 3)	3 (2, 4)	3 (3, 4)	4 (2, 4)	3 (2, 4)	3 (2, 3)	2 (1, 3)
Employer	3 (2, 3)	3 (1, 3)	3 (3, 4)	4 (3, 4)	2 (1, 4)	3 (2, 4)	2 (1, 3)	1 (1, 1)
Coworker	3 (2, 3)	2 (2, 3)	3 (2, 4)	3 (3, 4)	2 (1, 3)	3 (2, 4)	2 (1, 3)	1 (1, 2)
Support worker/disability service provider	2 (1, 3)	2 (1, 3)	3 (3, 4)	3 (2, 3)	2 (1, 3)	2.5 (2, 3)	2 (1, 3)	3 (2, 4)
Religious leaders	1 (1, 2)	1 (1, 2)	1 (1, 3)	2 (1, 3)	2 (1, 3)	1 (1, 3)	1 (1, 1)	1 (1, 2)
Community leaders	1 (1, 2)	1 (1, 2)	2 (1, 3)	2 (1, 3)	2 (1, 3)	1 (1, 3)	2 (2, 4)	1 (1, 3)
**Information about COVID-19, median (IQR)‡**								
Easy to understand?	4 (3, 4)	4 (3, 4)	4 (3, 4)	4 (3, 4)	4 (3, 4)	4 (3, 4)	3 (2, 4)	4 (3, 4)
Easy to find?	4 (3, 4)	4 (3, 4)	4 (3, 4)	4 (4, 4)	4 (3, 4)	4 (3, 4)	4 (3, 4)	4 (3, 4)
Relevant?	4 (3, 4)	4 (3, 4)	4 (3, 4)	4 (3, 4)	4 (3, 5)	4 (3, 4)	3 (3, 4)	3 (3, 4)

CI – confidence interval, IQR – interquartile range

*Not all characteristics sum to the overall sample due to missing data.

†Denotes a question that was not asked in the Aboriginal and Torres Strait Islander people survey. However, it is assumed that 100% of respondents were born in Australia.

‡Responses for exposure to COVID-19 information and trust items were: 1 = “Not at all”; 2 = “To a small extent”; 3 = “To a moderate extent”; and 4 = “To a great extent”. Responses for information about COVID-19 were: 1 = “Strongly disagree”; 2 = “Disagree”; 3 = “Neither agree nor disagree”; 4 = “Agree”; 5 = “Strongly agree”.

1092 respondents (76%) who responded to every item in the survey and were able to be involved in the latent class analysis. The latent class analysis with the lowest Akaike Information Criterion was the model with four classes. The marginal means for items in each class are outlined in Table S3 in the **Online Supplementary Document** and the variables that identified characteristics of each latent class are outlined in **Table 3**. The first class was named “COVID-safe mask wearers” and represented 10% of respondents. This class were generally supportive of COVID-safe behaviour and were more likely to wear a mask outdoors if they were unable to socially isolate. The demographic, COVID-19 information exposure, and trust variables associated with this class indicate that people in this class were more likely to be retired, concerned about COVID-19 infection, and trust COVID-19 information from celebrities/sportspeople, but not COVID-19 information from family and friends.

**Table 3:** Multivariable logistic regression models identifying defining characteristics of each latent class

Latent class	Variable	Odds ratio (95% CI)*
COVID-safe mask wearers	Retired	3.36 (1.51-7.48)
	Concern about getting COVID -19 infection	2.32 (1.13-4.77)
	Trust in COVID-19 information from celebrities/sportspeople	2.19 (1.16-4.13)
	Age	1.21 (1.06-1.39)
	Trust in COVID-19 information from family & friends	0.55 (0.36-0.86)
COVID-safe test takers	Concern about getting COVID-19 infection	2.62 (1.86-3.70)
	Belief that constant use of QR codes will reduce spread of COVID-19 in Australia	1.73 (1.28-2.34)
	Trust in COVID-19 information from news/TV/radio	1.69 (1.24-2.31)
	Trust in COVID-19 information from politicians	1.44 (1.02-2.03)
	Exposure to COVID-19 information from newspapers	1.44 (1.07-1.95)
	Exposure to COVID-19 information at work	1.36 (1.02-1.82)
	Trust in COVID-19 information from celebrities/sportspeople	0.47 (0.28-0.79)
	Concern about vaccine manufacturer motives	0.58 (0.42-0.79)
	Concern about COVID-19 restrictions	0.69 (0.51-0.92)
COVID-risk isolators	Concern about COVID-19 infection	0.41 (0.24-0.70)
	Support for QR codes	0.44 (0.31-0.62)
	Concern about COVID-19 infection if there were an outbreak	0.57 (0.39-0.83)
	Trust in COVID-19 information from politicians	0.63 (0.44-0.92)
	Trust in COVID-19 information from news/TV/radio	0.65 (0.45-0.94)
COVID-risk visitors	Deaf/hard of hearing subgroup	2.25 (1.04-4.87)
	Concern outbreak will affect physical health	1.63 (1.08-2.45)
	Concern for long-term vaccine side-effects	1.60 (1.09-2.34)
	Lives with dependents under 18 y of age	1.53 (1.06-2.21)
	Trust in COVID-19 information from celebrities/sportspeople	0.27 (0.16-0.45)
	Concern about COVID-19 infection	0.43 (0.26-0.70)
	Trust in COVID-19 information from news/TV/radio	0.54 (0.36-0.81)
	Female	0.61 (0.39-0.95)
	Belief that constant use of QR codes will reduce spread COVID-19 in Australia	0.64 (0.43-0.95)

CI – confidence interval

*Odds ratios >1 indicate the variable is more likely, and odds ratios <1 indicate the variable is less likely.

The second class was named “COVID-safe test takers” and represented 56% of the respondents. This class were generally supportive of COVID-safe behaviours and were more likely to get a COVID-19 test if they developed a cough than class 1, “COVID-safe mask wearers”. COVID-safe test takers were more concerned about getting COVID-19 infection and less about the impact of COVID-19 restrictions and the motives of COVID-19 vaccine manufacturers, were more likely to think that our community would be much less likely to spread COVID-19 if all of Australia always used QR codes when checking in to venues, have higher levels of trust in COVID-19 information from news programs on television and radio and politicians, lower levels of trust in COVID-19 information from celebrities/sportspeople, and were more exposed to COVID-19 information from newspapers and workplaces than other classes.

The third class was named “COVID-risk isolators” and represented 19% of the respondents. This class were generally not supportive of COVID-safe behaviours, though were not likely to visit people and attend public places if they developed a cough. This class was less concerned about getting COVID-19 infection both in general and if there was an outbreak. They reported lower likelihood of using QR codes when checking in to venues. They were less trusting of COVID-19 information from politicians and news programs on television or radio.

The fourth class was named “COVID-risk visitors” and represented 15% of the respondents. This class were generally not supportive of COVID-safe behaviour but were more likely to visit people and attend public places if they developed a cough than the “COVID-risk isolators”. Respondents in this class were less likely to be female, more likely to live with dependents under 18 years of age, more likely to come from our deaf/hard of hearing vulnerable group, have higher levels of concern that a COVID-19 outbreak in the next month would affect their own physical health, higher levels of concern about the potential long-term impacts of COVID-19 vaccines, lower levels of trust in COVID-19 information from news programs on television or radio and from celebrities/sportspeople, and less likely to think that our community would be much less likely to spread COVID-19 if all of Australia always used QR codes when checking in to venues.

**Table 4** describes response patterns between latent classes across the items asking if COVID-19 information in Australia was “easy to understand”, “easy to find”, and “relevant to you”, while **Table 5** outlines the comparisons between the vulnerable subgroups and the general community sample.

**Table 4:** Univariable ordered logistic regression models examining the association between latent classes about whether COVID-19 information is easy to understand, easy to find, and relevant

		Odds ratio and 95% CI*		
**Right now, do you feel that information about COVID -19 here in Australia is…**	**Median (IQR); mean (SD)**	**COVID-safe mask wearers**	**COVID-safe test takers**	**COVID-risk isolators**	
**Easy to understand?**					
COVID-safe mask wearers	4 (3, 4); 3.4 (1.1)	-	-	-	
COVID-safe test takers	4 (3, 4); 3.5 (1.0)	0.95 (0.64, 1.39)	-	-	
COVID-risk isolators	3 (2, 4); 3.1 (1.1)	1.97 (1.26, 3.06)†	2.09 (1.56, 2.81)†	-	
COVID-risk visitors	3 (2, 4); 3.2 (1.2)	1.62 (1.03, 2.54)†	1.77 (1.29, 2.44)†	0.85 (0.59, 1.24)	
**Easy to find?**					
COVID-safe mask wearers	4 (3, 4); 3.6 (1.1)	-	-	-	
COVID-safe test takers	4 (3, 4); 3.7 (0.9)	0.85 (0.57, 1.27)	-	-	
COVID-risk isolators	4 (3, 4); 3.4 (1.1)	1.48 (0.95, 2.32)	1.77 (1.31, 2.39)†	-	
COVID-risk visitors	4 (3, 4); 3.4 (1.1)	1.62 (1.03, 2.54)†	1.63 (1.17, 2.26)†	0.93 (0.63, 1.36)	
**Relevant?**					
COVID-safe mask wearers	4 (3, 4); 3.6 (1.0)	-	-	-	
COVID-safe test takers	4 (3, 4); 3.7 (0.9)	0.82 (0.55, 1.22)	-	-	
COVID-risk isolators	3 (2, 4); 3.1 (1.1)	2.30 (1.48, 3.58)†	2.96 (2.18, 4.00)†	-	
COVID-risk visitors	4 (3, 4); 3.4 (1.1)	1.38 (0.88, 2.16)	1.73 (124, 2.40)†	0.61 (0.42, 0.90)†	

CI – confidence interval, SD – standard deviation

*Odds ratios >1 indicate the classes are different, and odds ratios <1 indicate the classes are similar. Responses for information about COVID -19 were: 1 = “Strongly disagree”; 2 = “Disagree”; 3 = “Neither agree nor disagree”; 4 = “Agree”; 5 = “Strongly agree”.

†*P*-value <0.05.

**Table 5:** Comparison of response to current COVID-19 information between vulnerable communities and the general community

Right now, do you feel that information about COVID -19 here in Australia is…	Vulnerable communities	Median (IQR)	Odds ratio (95% CI)*
**Easy to understand?**	Aboriginal and Torres Strait Islander people	3 (2, 4)	0.79 (0.47-1.32)
	Aged care workers	4 (3, 4)	1.74 (1.03-2.95)
	People with a disability and their carers	4 (3, 4)	1.31 (0.78-2.21)
	Deaf/hard of hearing	4 (3, 4)	1.49 (0.90-2.47)
	Street-based sex workers	4 (3, 4)	1.40 (0.82-2.40)
	Refugee and asylum seekers	4 (3, 4)	2.03 (1.19-3.47)
	General community sample	4 (3, 4)	Reference
**Easy to find?**	Aboriginal and Torres Strait Islander people	4 (3, 4)	0.91 (0.53-1.56)
	Aged care workers	4 (4, 4)	1.53 (0.89-2.61)
	People with a disability and their carers	4 (3, 4)	1.07 (0.63-1.83)
	Deaf/hard of hearing	4 (3, 4)	0.73 (0.45-1.21)
	Street-based sex workers	4 (3, 4)	1.23 (0.71-2.13)
	Refugee and asylum seekers	4 (3, 4)	1.65 (0.95-2.86)
	General community sample	4 (3, 4)	Reference
**Relevant to you?**	Aboriginal and Torres Strait Islander people	3 (3, 4)	0.59 (0.36-0.98)
	Aged care workers	4 (3, 4)	1.75 (1.03-2.97)
	People with a disability and their carers	4 (3, 4)	1.44 (0.84-2.45)
	Deaf/hard of hearing	4 (3, 4)	1.03 (0.61-1.72)
	Street-based sex workers	3 (3, 4)	0.79 (0.46-1.35)
	Refugee and asylum seekers	4 (3, 5)	2.15 (1.24-3.74)
	General community sample	4 (3, 4)	Reference

CI – confidence interval, SD – standard deviation

*Odds ratios >1 indicate variable is more likely, and odds ratios <1 indicate variable is less likely. Responses for information about COVID -19 were: 1 = “Strongly disagree”; 2 = “Disagree”; 3 = “Neither agree nor disagree”; 4 = “Agree”; 5 = “Strongly agree”.

There were differences between latent classes regarding how easy they found COVID-19 information to understand. COVID-risk visitors and COVID-risk isolators thought COVID-19 information was more difficult to understand than COVID-safe mask wearers and COVID-safe test takers. Vulnerable communities were largely similar to the general community, except for aged care workers and refugee and asylum seekers, who thought COVID-19 information was easier to understand. COVID-risk visitors and COVID-risk isolators thought COVID-19 information was more difficult to find than COVID-safe test takers, and COVID-risk visitors thought COVID-19 information was more difficult to find than COVID-safe mask wearers. Vulnerable communities were similar to the general community in terms of finding COVID-19 information.

There were differences in the perceived relevance of COVID-19 information observed between the latent classes. COVID-risk isolators were more than two times more likely to find COVID-19 information less relevant than COVID-safe mask wearers and COVID-safe test takers. COVID-risk visitors found COVID-19 information less relevant than COVID-safe test takers and were similar to COVID-risk isolators. Vulnerable communities were largely similar to the general community, apart from Aboriginal and Torres Strait Islander people, who thought COVID-19 information was less relevant to them than the general community, and aged care workers and refugee and asylum seekers, who thought COVID-19 information was more relevant to them than the general community.

The demographic (including vulnerable community membership) and latent class variables that were independently associated with whether people found COVID-19 information easy to understand, easy to find and relevant to them are presented in **Table 6**.

**Table 6:** Multivariable ordered logistic regression models of variables associated with whether people find COVID-19 information easy to understand, easy to find, and relevant to them

Right now, do you feel that information about COVID -19 here in Australia is…	Variable	Odds ratio (95% CI)*
**Easy to understand?**	Employed across multiple jobs	1.71 (1.19-2.46)
	Single/never married	1.48 (1.07-2.05)
	COVID-risk isolators	0.50 (0.36-0.69)
	COVID-risk visitors	0.59 (0.42-0.84)
**Easy to find?**	Employed across multiple jobs	1.74 (1.15-2.64)
	Unable to read well	0.40 (0.17-0.94)
	COVID-risk visitors	0.57 (0.40-0.81)
	COVID-risk isolators	0.57 (0.41-0.80)
	Other employment situation	0.58 (0.36-0.92)
	Not in paid work but works in unpaid roles (ie, caring)	0.58 (0.35-0.97)
**Relevant to you?**	COVID-safe test takers	1.47 (1.08-1.97)
	Aboriginal and Torres Strait Islander People	0.39 (0.21-0.72)
	Street-based sex workers	0.52 (0.29-0.93)
	COVID-risk isolators	0.54 (0.37-0.78)

CI – confidence interval

*Odds ratios >1 indicate variable is more likely, and odds ratios <1 indicate variable is less likely.

Variables independently and positively associated with COVID-19 information being easy to understand were being employed across multiple jobs and being single or never married, while being in either of the two COVID-risk classes was negatively associated. The only variable positively associated with COVID-19 information being easy to find was being employed across multiple jobs, while being unable to read well, in either of the COVID-risk classes, having an “other” employment situation (eg, maternity leave), or not being in paid work but working in an unpaid work role were negatively associated. The only variable positively associated with COVID-19 information being “relevant to them” was being in the COVID-safe test taker latent class, while people who were Aboriginal and Torres Strait Islander Peoples, street-based sex workers, or in the COVID-risk isolator latent class were negatively associated.

## DISCUSSION

The “vulnerable groups” examined in this study generally found COVID-19 public health messaging to be as easy, if not easier, to find, understand, and to be relevant to them as the general community. This suggests that public health messaging in Australia, both in general and that specifically targeted at these groups, may have largely been successful. However, there were two “COVID risk” latent sub-groups that cut across both vulnerable groups and the general community who could possibly be candidates for additional public health messaging. These two “COVID risk” groups made up 34% of our respondents and were less likely to report that COVID-19 public health information was easy to find or easy to understand. They had lower levels of trust in COVID-19 information presented in news programs on the television or radio, which are key sources of COVID-19 information for the general public [36]. This indicates that we need to reconsider how this information is being presented to them via these media or pursue other strategies to try to influence their views.

Our findings are similar to those from a study that used latent profile analysis from an international sample of 1575 participants [37]. This study found two separate profiles based on COVID-safe behaviour recommendations; people who are compliant with COVID-19 recommendations and people who are not. There are several explanations for why people might ignore COVID-safe health recommendations, such as distrust [38,39], anger [40,41], misinformation [42], socioeconomic and/or occupational circumstances, backgrounds, and choices [17], and certain psychological traits [43]. Overcoming these factors to embed COVID-safe behaviours as a normal part of life will require varied approaches, and public health messaging specific to the needs of these COVID-risk classes will need to be considered. Indeed, the COVID-risk classes in our study found COVID-19 information was less relevant, easy to find, and easy to understand than COVID-safe classes. These findings may be explained by people’s attitudes and behaviours about COVID-19 being influenced by health literacy (ie, COVID-19 information is not easy to understand) [42], and that people who are less likely to comply with COVID-19 restrictions check official COVID-19 information less frequently (ie, COVID-19 information is not easy to find) and have less trust in information sources (ie, COVID-19 information is not relevant) [37].

Instances where our vulnerable communities reported more positive responses on ratings of COVID-19 information accessibility, relevance, and ease of understanding than our general community sample were present for aged care workers and refugee and asylum seekers. A possible explanation for the more positive response from aged care workers is that this group were targeted with highly specific messaging tailored to their group’s needs, particularly given the prominence of early outbreaks in Australian aged care facilities [44]. Refugee and asylum groups have also been the focus of targeted messaging approaches in the state of Victoria in Australia [45]. However, Aboriginal and Torres Strait Islander people and street-based sex workers were less likely to find current COVID-19 messaging relevant to them but were not independently more likely to find COVID-19 information harder to find or understand. These findings indicate that there are still vulnerable communities that may benefit from further tailoring of COVID-19 public health messaging to be relevant to their circumstances. In contrast to these somewhat inconsistent findings, the latent COVID-risk classes were consistently identified as having poorer responses to current COVID-19 messaging in Australia. It may be that public health efforts are better targeted at the latent COVID-risk classes that cut across both our general and vulnerable communities, than targeted at the vulnerable communities themselves. Further research comparing responses of people within the vulnerable communities and those in the latent COVID-risk classes to different messaging approaches is needed.

There are some important limitations we now highlight to help with the interpretation of the study findings. First, in multiple regression models, some variables provide similar information to other variables during model development (multicollinearity), leading to only one of these variables being retained in the model. For example, people from the deaf/hard of hearing vulnerable group were more likely to be present in the latent COVID-risk visitors class. Thus, the presence of the latent COVID-risk visitor class in our multiple regression models examining accessibility, relevance and ease of understanding COVID-19 information may have obscured the importance of the deaf/hard of hearing group in these outcomes. Second, the data were cross-sectional, making directional causality difficult to establish. For example, we cannot say that trust in COVID-19 information from politicians causes changes in behaviour or attitudes to COVID-19. On one hand, trust in COVID-19 information from a particular source may influence their COVID-19 attitudes and behaviours. However, it may be that COVID-19 attitudes and behaviours drive people to seek COVID-19 information from particular sources.

These findings should prompt further research to establish whether tailored public messaging can effectively change behaviours and attitudes to those that align with COVID-safe health recommendations. We found differences between people who accept and people who reject COVID-safe behaviours, which can be targeted to improve public messaging about COVID-19. These differences include the people/groups who are trusted to deliver COVID-19 information, and whether COVID-19 information is relevant, easy to find and easy to understand. Additionally, singular mass media campaigns that are not tailored to specific people/groups could have unanticipated negative consequences in particular segments of the community who are arguably in need of change, which requires further exploration. Finally, further prospective research that evaluates the similarities and differences between vulnerable communities and the general community regarding COVID-19 information can inform future public health messaging campaigns.

## CONCLUSIONS

Latent groups representing a significant segment of the community are not receptive to key COVID-safe health recommendations. The information needs of these latent groups are not being met by current public health messaging. In contrast, vulnerable communities – who may be considered to require greater public health assistance – have a similar, and for some subgroups, a better understanding of COVID-19 information than the general community. This suggests that either current public health messaging – or targeted strategies – for some vulnerable communities has been effective, or alternatively, that their attitudes to specific COVID-19 safe behaviours were better from the outset. Further research is needed to identify and evaluate tailored COVID-19 messaging strategies that can effectively shift attitudes and behaviours to increase the proportion of the population engaging in COVID-safe behaviours.

## Additional material


Online Supplementary Document
