# Supplementary material for: Australian public health COVID-19 messaging is missing its mark in some vulnerable communities and people who reject COVID-19 safety advice
Source: J Glob Health. 2022 Sep 3;12:05037. doi: 10.7189/jogh.12.05037 (PMC9441129; doi:10.7189/jogh.12.05037)
Supplement: Online Supplementary Document [file jogh-12-05037-s001.pdf]

ONLINE SUPPLEMENTARY DOCUMENT

Title: Australian public health COVID-19 messaging is missing its mark in some vulnerable communities and people who reject COVID-19 safety advice

Authors: Megan Jepson, Glen A Whittaker, Lauren Robins, Katrina M Long, Cylie M Williams, Grant Russell, Keith D Hill, Libby Callaway, Jim Hlavac, Louisa Willoughby, Terry P Haines

Table S1. Recruitment methods.

Sub-group	Recruitment method
Community	An invitation to participate in the survey was advertised on Facebook, Instagram and Twitter. A direct link to the survey was included in the advertisement where potential participants were directed to complete the survey upon consenting.
People with a disability and their carers	A one page easy to read study overview was advertised at key Specialist Disability Accommodation and Day Program sites throughout the participating organisations. As an honorarium to participate was provided, potential participants were invited to express interest in participating in the survey by providing their contact details so that they could be provided with the link to the survey.
Aged Care workers	A one page easy to read study overview was advertised throughout the participating organisation. As an honorarium to participate was provided, potential participants were invited to express interest in participating in the survey by providing their contact details so that they could be provided with the link to the survey.
Refugee and asylum seekers	On-site bi-cultural workers assisted recruitment of Dari-speaking refugee and asylum seekers and Sinhalese-speaking refugee and asylum seekers. The survey was advertised through community organisation's contacts and networks using a short, easy-to-read outline of the study project. Sinhalese speaking participants completed the survey online via Qualtrics, while Dari speaking participants completed a paper-based survey with the assistance of a bi-cultural worker.
Deaf/hard of hearing	An easy-to-read study overview was advertised on the participating organisation's Facebook page – as well as cognate organisations in other states. The post included an Auslan version of the call for participants in a linked video. A direct link to the survey was included in the advertisement where potential participants were directed to complete the survey upon consenting. The main Qualtrics survey itself also included Auslan videos outlining the questions that participates could choose whether to view.
Aboriginal Torres Strait islanders	Participants were recruited onsite at local meeting places with assistance from Peninsula Health's Aboriginal health worker and liaison officer using a one page easy to read study overview information sheet. Consenting participants then completed a paper-based survey, supported by the Aboriginal health worker or meeting place staff if needed.
Street-based sex workers	Participants were recruited on site, in person via word of mouth. iPads were issued so that those who consented to participate could complete the survey (with assistance from the organisation's staff members) online using Qualtrics.

Appendix S2. Survey questions.

Demographics ... **3**

Q1 Who is completing this survey? ... 4

Q2 What country were you born in? ... 4

Q3 What year did you first arrive in Australia? ... 4

Q4 Do you identify as Aboriginal or Torres Strait Islander? ... 4

Q5 What is your religion? ... 5

Q6 How often do you attend religious services? ... 5

Q7 Please select your age bracket ... 6

Q8 Please select your gender ... 6

Q9 How well do you feel you read English? ... 7

Q10 Do you prefer to read things in a language other than English? ... 7

Q11 How well do you read in your preferred language? ... 7

Q12 Please select the option that best describes your current employment/ study situation ... 8

Q13 Please select the option that best describes your current marital status ... 8

Q14 What is the postcode where you usually live? ... 9

Q15 Do you have a current permanent address? ... 9

Q16 Please select the option that best describes your current living arrangement ... 9

Q17 How many people live at your current residence (do not count yourself)? ... 10

Q18 Please indicate the extent to which you have been exposed to information about COVID-19 in the past month from ... 11

Q19 Please indicate how much you trust the information about COVID-19 from the following sources: ... 14

Q20 Right now, do you feel that information about COVID-19 here in Australia is: ... 16

Q21 If you disagreed or strongly disagreed to any of the above questions, give reasons why ... 16

Vaccine ... **17**

Q22 At present, how likely are you to: ... 17

Q23 Have you had the COVID-19 vaccine? (1 or both doses if applicable) ... 18

Q24 How likely would you be to have a COVID-19 vaccine right away, if a vaccine was available to you right now at no cost (it is currently free in Australia)? ... 18

Q25 If you **haven't had** the vaccine, we would like you to imagine that you did have the vaccine. We would now like you to say how likely you are to (answer all of the following questions): ... 19

Q26 In regards to the vaccine, how concerned are you about ... 20

Q27 Give your level of agreement to the following statements: ... 21

Q28 How would you prefer to receive information about the COVID-19 vaccination? ... 22

Concern about getting COVID ... **22**

Q29 How concerned are you that in the next month ... 22

Q30 Please indicate the extent of your concern that COVID-19 may affect your life in the next month in terms of 24
Q31 We now want you to imagine that there has been a community outbreak of COVID-19. Imagine that this was a case of community transmission that happened in your suburb. In this case, how concerned would you be that in the next month 25
Q32 Continue to imagine there has been an outbreak of COVID-19 in your suburb. Please indicate the extent of your concern that COVID-19 may affect your life in the next month in terms of 26
Symptoms 27
Q33 If you were to develop a **fever** (but that was the only health concern), how likely would you be to 28
Q34 If you were to develop a **cough** (but that was the only issue), how likely would you be to 29
Q35 How concerned are you about the following things when you get tested for COVID-19? 30
Hot spots 31
Q 36 In this imaginary scenario, how likely would it be that in the next 24 hours you would 31
Q37 How would you prefer to receive updates about what "hot spots" have been identified? 32
Q38 How would you prefer to receive information about what people need to do if they have visited an "exposure site"? 33
QR Codes 33
Q39 When a QR code or venue check-in service is provided at a venue, how often do you use it to check in? 34
Q40 In regards to checking in with a QR code 34
Q41 Please let us know any further comments or questions you may have about what has been asked in this survey 35
Q42 List of disabilities question added to disability and community survey only 36
List of Countries for Q2 37

☐ Demographics

Q1 Who is completing this survey?

○ I am completing the survey for myself ○ I am completing the survey with the help of another individual (e.g. family, health/support worker) ○ I am a completing the survey on behalf of the person

---

Q2 What country were you born in?

▼ Afghanistan (1) ... Zimbabwe (1357)

Australia= 9

*See additional document (List of Countries) for all other country scores.

Q3 What year did you first arrive in Australia?

____

Q4 Do you identify as Aboriginal or Torres Strait Islander?

- ☐ No
- ☐ Yes, Aboriginal

Yes, Torres Strait Islander

Yes, both Aboriginal and Torres Strait Islander

Q5 What is your religion?

No religion Catholic Orthodox Other Christian Buddhism Islam

Hinduism Sikhism Judaism Other (please specify below)

____

Q6 How often do you attend religious services?

Weekly Monthly

Less than monthly

Only for special occasions

Never

Q7 Please select your age bracket

○ Less than 30 years ○ 30-39 years ○ 40-49 years ○ 50-59 years ○ 60-69 years ○ 70-79 years

○ 80 years or more

Q8 Please select your gender

○ Male ○ Female ○ Non-binary / gender diverse ○ Prefer not to say ○ My gender isn't listed. I identify as (please specify below how you choose to identify) ____

☐

Q9 How well do you feel you read English?

○ Very well ○ Well ○ Not well

○ I do not read English at all

☐ Q10 Do you prefer to read things in a language other than English?

○ No

○ Yes, please state below

____

☐ Q11 How well do you read in your preferred language?

○ Very well ○ Well ○ Not well

○ I do not read above language at all

Disability Q should be here for some surveys (community group survey and disability survey)-
Question

Q12 Please select the option that best describes your current employment/ study situation

- I am a student
- I am employed full time in one job
- I am employed part time in one job
- I am employed as a casual in one job
- I am employed across multiple jobs
- I am self-employed
- I am not in paid employment, but work in unpaid roles (eg. I am a care-giver)
- I am unemployed but looking for work
- I am retired
- I am not seeking employment
- I am unable to work due to my disability/health condition
- Other (please describe below) ____

Q13 Please select the option that best describes your current marital status

- Married or de facto
- Widowed
- Divorced
- Separated but not divorced
- Single or never married
- Other (please specify below) ____

Q14 What is the postcode where you usually live?

____

Q15 Do you have a current permanent address?

○ Yes ○ No

Q16 Please select the option that best describes your current living arrangement.

○ Staying with friends or family (e.g. couch surfing) ○ Government housing ○ Crisis or emergency accommodation ○ Boarding house ○ Sleeping rough on the streets

○ Other (please describe below)

____

Q17 How many people live at your current residence (do not count yourself)?

If you live in a block of apartments, units or similar, just specify the number who live in your particular apartment or unit. (Click and slide EVERY slider to the correct number)

	0	1	2	3	4	5	6	7	8	9	10
...who are 2 years old or younger											
...who are between 3 and 12 years old											
...who are between 13 and 17 years old											
...who are between 18 and 64 years old											
...who are 65 years or older											

There are many potential sources of information about COVID-19 and how we should respond to it.

Q18 Please indicate the extent to which you have been exposed to information about COVID-19 in the past month from...

(Every response must be clicked to progress)

	Not at all	To a small extent	To a moderate extent	To a great extent
Newspapers	○	○	○	○
Radio	○	○	○	○
Television - news programs	○	○	○	○
Television - other programs	○	○	○	○
Facebook feeds	○	○	○	○
Facebook private groups	○	○	○	○
Instagram	○	○	○	○
Twitter	○	○	○	○
TikTok	○	○	○	○
Online blogs	○	○	○	○
Podcasts	○	○	○	○
General browsing on the internet	○	○	○	○
Australian Government "Coronavirus Australia" App	○	○	○	○

Other web-pages and resources specifically prepared by the Australian Government

Workplace

Friends and family

Community or religious gatherings

Notice boards

Other sources (please specify below)

☐ Q19 Please indicate how much you trust the information about COVID-19 from the following sources:

	Not at all	To a small extent	To a moderate extent	To a great extent	Not applicable
Australian Politicians	○	○	○	○	○
Australian Federal or state chief medical officers	○	○	○	○	○
Scientists	○	○	○	○	○
Your doctor/General Practitioner	○	○	○	○	○
Other health care professional	○	○	○	○	○
Support worker/disability service provider	○	○	○	○	○
Your religious leader e.g. Priest, Rabbi etc.	○	○	○	○	○
Community leaders e.g. elders etc.	○	○	○	○	○
Family	○	○	○	○	○
Friends	○	○	○	○	○
Your employer	○	○	○	○	○
Other coworkers/people who work in your industry	○	○	○	○	○
News programs on TV and radio	○	○	○	○	○

News on social media	○	○	○	○	○
Celebrities/TV personalities/ elite sportspeople	○	○	○	○	○

Q20 Right now, do you feel that information about COVID-19 here in Australia is:

	Strongly disagree	Disagree	Neither agree nor disagree	Agree	Strongly Agree
Easy to understand	○	○	○	○	○
Easy to find	○	○	○	○	○
Relevant to you	○	○	○	○	○

Q21 If you disagreed or strongly disagreed to any of the above questions, give reasons why.

____

End of Block: Demographics

Vaccine

Q22 At present, how likely are you to:

	Extremely unlikely	Unlikely	Neutral	Likely	Extremely likely
Wear a mask when required by government	○	○	○	○	○
Wear a mask inside when in a public place e.g. a shop or medical clinic	○	○	○	○	○
Wear a mask outside when you are unable to socially distance	○	○	○	○	○
Socially distance in public places	○	○	○	○	○
Socially distance with friends or family members	○	○	○	○	○
Attempt international travel	○	○	○	○	○

Q23 Have you had the COVID-19 vaccine? (1 or both doses if applicable)

○ Yes ○ No

Q24 How likely would you be to have a COVID-19 vaccine right away, if a vaccine was available to you right now at no cost (it is currently free in Australia)?

	Very unlikely	Very likely
	0 1 2 3 4 5 6 7 8 9 10	
()	The image part with relationship ID rId8 was not found in the file.	

Q25 If you haven't had the vaccine, we would like you to imagine that you did have the vaccine. We would now like you to say how likely you are to (answer all of the following questions):

	Extremely unlikely	Unlikely	Neutral	Likely	Extremely likely
Wear a mask when required to by government	○	○	○	○	○
Wear a mask inside when in a public place e.g. a shop or medical clinic	○	○	○	○	○
Wear a mask outside when you are unable to socially distance	○	○	○	○	○
Socially distance in public places	○	○	○	○	○
Socially distance with friends or family members	○	○	○	○	○
Attempt international travel	○	○	○	○	○

Q26 In regards to the vaccine, how concerned are you about...

	Not at all concerned	Slightly concerned	Somewhat concerned	Moderately concerned	Extremely concerned
...the Oxford/Astrazeneca vaccine	○	○	○	○	○
...the Pfizer/BioNTech vaccine	○	○	○	○	○
...immediate sideeffects/safety of the vaccine	○	○	○	○	○
...potential longterm side effects/safety of the vaccine	○	○	○	○	○
...approval process of the vaccine	○	○	○	○	○
...how the vaccine was developed	○	○	○	○	○
...the Government's motivation to develop the vaccine	○	○	○	○	○
...the drug companies' motivation to develop the vaccine	○	○	○	○	○

Q27 Give your level of agreement to the following statements:

	Strongly disagree	Disagree	Neither agree nor disagree	Agree	Strongly agree
If **all Australians** were vaccinated against COVID-19, **our community** would be much less likely to spread COVID-19	○	○	○	○	○
If **I** had the COVID-19 vaccine, **I** would be much less likely to get COVID-19.	○	○	○	○	○

The image part with relations

☐

Q28 How would you prefer to receive information about the COVID-19 vaccination?

☐ Messaging on social media

☐ Press conference on news/TV/radio

☐ Newspapers

☐ Information on government web-sites

☐ Messages sent to my mobile phone

☐ Messages sent to me from my place of work/education

☐ Other. Please specify ____

End of Block: Vaccine

Concern about getting COVID

☐ Q29 How concerned are you that in the next month...

	Not concerned at all	A little concerned	Somewhat concerned	Very concerned	Extremely concerned

You will get COVID-19	○	○	○	○	○
You will give COVID-19 to someone else	○	○	○	○	○
You will be impacted by government restrictions designed to prevent the spread of COVID-19	○	○	○	○	○
Someone you care about will get COVID-19	○	○	○	○	○
Someone you care about will be impacted by government restrictions designed to prevent the spread of COVID-19	○	○	○	○	○

Q30 Please indicate the extent of your concern that COVID-19 may affect your life in the next month in terms of...

	Not at all concerned	A little concerned	Somewhat concerned	Very concerned	Extremely concerned
Your physical health	○	○	○	○	○
Your mental health	○	○	○	○	○
Your ability to work	○	○	○	○	○
Your ability to purchase food	○	○	○	○	○
Your ability to get places you need to go	○	○	○	○	○
Your ability to visit friends or family/socialise	○	○	○	○	○

Q31 We now want you to imagine that there has been a community outbreak of COVID-19. Imagine that this was a case of community transmission that happened in your suburb. In this case, how concerned would you be that in the next month...

	Not at all concerned	A little concerned	Somewhat concerned	Very concerned	Extremely concerned
You would get COVID-19	○	○	○	○	○
You would give COVID-19 to someone else	○	○	○	○	○
You would be impacted by government restrictions designed to prevent the spread of COVID-19	○	○	○	○	○
Someone you care about would get COVID-19	○	○	○	○	○
Someone you care about would be impacted by government restrictions designed to prevent the spread of COVID-19	○	○	○	○	○

☐

Q32 Continue to imagine there has been an outbreak of COVID-19 in your suburb. Please indicate the extent of your concern that COVID-19 may affect your life in the next month in terms of...

	Not at all concerned	A little concerned	Somewhat concerned	Very concerned	Extremely concerned
Your own physical health	○	○	○	○	○
Your own mental health	○	○	○	○	○
Your ability to work	○	○	○	○	○
Your ability to purchase food	○	○	○	○	○
Your ability to get places you need to go	○	○	○	○	○
Your ability to visit friends or family/socialise	○	○	○	○	○

☐

End of Block: Concern about getting COVID

☐ Symptoms

The following questions relate to health issues you might experience in the coming weeks or months.
These are common issues that you are likely to have previously experienced.

We are interested in understanding how you would react if you developed these symptoms.

Q33 If you were to develop a fever (but that was the only health concern), how likely would you be to...

	Extremely unlikely	Unlikely	Neutral	Likely	Extremely likely	Not applicable
...tell other people who you may have been in close contact with in the previous 2 days e.g. people you live with	○	○	○	○	○	○
...tell relevant people at your place of work	○	○	○	○	○	○
...seek an appointment with your general practitioner	○	○	○	○	○	○
...seek testing specifically for COVID-19	○	○	○	○	○	○
...visit public places e.g. shopping centre	○	○	○	○	○	○
...spend time with people you **do not live with**	○	○	○	○	○	○

...spend time with people you **do live with**						

Q34 If you were to develop a cough (but that was the only issue), how likely would you be to...

	Extremely unlikely	Unlikely	Neutral	Likely	Extremely likely	Not applicable
...tell other people who you may have been in close contact with in the previous 2 days e.g. people you live with						
...tell relevant people at your place of work						
...seek an appointment with your general practitioner						
...seek testing specifically for COVID-19						
...visit public places e.g. shopping centre						

...spend time with people you **do not live with**	○	○	○	○	○	○
...spend time with people you **do live with**	○	○	○	○	○	○

Q35 How concerned are you about the following things when you get tested for COVID-19?

	Not at all concerned	A little concerned	Somewhat concerned	Very concerned	Extremely concerned
The time it takes waiting in line	○	○	○	○	○
The pain experienced during a test	○	○	○	○	○
The time it takes to get your results back	○	○	○	○	○
Having to isolate while waiting for your results	○	○	○	○	○
Not being able to work while waiting for results	○	○	○	○	○

End of Block: Symptoms

Hot spots

There have been some COVID-19 outbreaks in Australia. The government has reported locations that people with COVID-19 have visited, known as "exposure sites" or "hot spots".

We want you to imagine that:

You visited a "hot spot" 2 days ago while an infected person was there,
You have not developed COVID-19 symptoms,
You do not know if you are infected and you have not been tested.

Q 36 In this imaginary scenario, how likely would it be that in the next 24 hours you would...

	Extremely unlikely	Unlikely	Neutral	Likely	Extremely likely	Not applicable
...tell other people who you may have been in close contact with in the previous 2 days e.g. people you live with	○	○	○	○	○	○
...tell relevant people at your place of work	○	○	○	○	○	○
...seek an appointment with your general practitioner	○	○	○	○	○	○
...seek testing specifically for COVID-19	○	○	○	○	○	○

...visit public places e.g. shopping centre	○	○	○	○	○	○
...spend time with people you **do not live with**	○	○	○	○	○	○
...spend time with people you **do live with**	○	○	○	○	○	○

Q37 How would you prefer to receive updates about what "hot spots" have been identified?

- ☐ Messaging on social media
- ☐ Press conferences on news/TV/radio
- ☐ Newspapers
- ☐ Information on government web-sites
- ☐ Message sent to my mobile phone
- ☐ Messages from my place of work
- ☐ Other. Please specify ____

Q38 How would you prefer to receive information about what people need to do if they have visited an "exposure site"?

- Messaging on social media
- Press conferences on news/TV/radio
- Newspapers
- Information on government web-sites
- Message sent to my mobile phone
- Messages from my place of work
- Other. Please specify
- ____

End of Block: Exposure sites

QR Codes

Many State Governments have set up a QR Code or check-in service for venues to keep a record of visitors. If a venue has the QR code sign, you scan the barcode (like the one below) with your mobile phone camera or log in using the written instructions. You are then requested to provide your name and phone number.

Q39 When a QR code or venue check-in service is provided at a venue, how often do you use it to check in?

○ Never ○ Rarely ○ Sometimes ○ Often

○ Always

Q40 In regards to checking in with a QR code...

	Strongly disagree	Disagree	Neither agree nor disagree	Agree	Strongly agree
If **all of Australia** always used QR codes checking in to venues, **our community** would be much less likely to spread COVID-19	○	○	○	○	○
If **I** always used QR codes checking in to venues,**I** would be much less likely to get COVID-19	○	○	○	○	○

End of Block: QR Code checking in

Start of Block: End of survey

Q41 Please let us know any further comments or questions you may have about what has been asked in this survey.

____

Please click next to submit your details.

Next

End of Block: End of survey

☐ Q42 List of disabilities question added to disability and community survey only

Do any of the following apply to you? Please select any that apply

- ☐ I have an acquired brain injury
- ☐ I have autism
- ☐ I am blind or I have a vision impairment
- ☐ I have cerebral palsy
- ☐ I have down syndrome
- ☐ I am deaf or hard of hearing
- ☐ I have an intellectual disability
- ☐ I have multiple sclerosis
- ☐ I have a psychological disability
- ☐ I had a stroke
- ☐ I have a spinal cord injury
- ☐ I have another neurological disability
- ☐ I have a physical disability
- ☐ I have a sensory or speech disability
- ☐ I have a different disability. Please list below

____

☐ List of Countries for Q2

What country were you born in?

Afghanistan

Albania

Algeria Andorra

Angola

Antigua and Barbuda

Argentina

Armenia Australia

Austria

Azerbaijan

Bahamas Bahrain

Bangladesh

Barbados Belarus

Belgium

Belize

Benin

Bhutan

Bolivia Bosnia and Herzegovina

Botswana

Brazil

Brunei Darussalam

Bulgaria

Burkina Faso

Burundi

Cambodia

Cameroon

Canada

Cape Verde

Central African

Republic Chad

Chile China

Colombia Comoros

Congo, Republic of

the...

Costa Rica

Côte d'Ivoire

Croatia

Cuba

Cyprus Czech Republic

Democratic People's Republic of Korea

Democratic Republic of the Congo

Denmark Djibouti

Dominica Dominican Republic

Ecuador Egypt El Salvador

Equatorial Guinea Eritrea

Estonia Ethiopia Fiji

Finland

France

Gabon

Gambia

Georgia

Germany Ghana

Greece Grenada

Guatemala

Guinea Guinea-Bissau

Guyana Haiti

Honduras Hong

Kong (S.A.R.) Hungary

Iceland India

Indonesia Iran,

Islamic Republic of...

Iraq

Ireland

Israel

Italy

Jamaica

Japan

Jordan

Kazakhstan

Kenya

Kiribati

Kuwait Kyrgyzstan

Lao People's

Democratic Republic

Latvia Lebanon

Lesotho Liberia

Libyan Arab

Jamahiriya

Liechtenstein

Lithuania

Luxembourg

Madagascar

Malawi

Malaysia

Maldives
Mali

Malta Marshall Islands

Mauritania Mauritius

Mexico Micronesia,

Federated States of...

Monaco

Mongolia

Montenegro

Morocco

Mozambique

Myanmar Namibia

Nauru Nepal

Netherlands

New Zealand

Nicaragua

Niger

Nigeria

North Korea

Norway

Oman Pakistan

Palau

Panama Papua

New Guinea

Paraguay Peru

Philippines

Poland Portugal

Qatar

Republic of Korea

Republic of Moldova

Romania

Russian Federation

Rwanda

Saint Kitts and Nevis

Saint Lucia

Saint Vincent and the Grenadines
Samoa

San Marino Sao

Tome and Principe

Saudi Arabia

Senegal Serbia

Seychelles

Sierra Leone

Singapore Slovakia

Slovenia

Solomon Islands

Somalia South

Africa South Korea

Spain Sri Lanka

Sudan

Suriname

Swaziland

Sweden

Switzerland

Syrian Arab Republic Tajikistan

Thailand The former Yugoslav Republic of

Macedonia Timor-Leste Togo Tonga

Trinidad and Tobago Tunisia Turkey

Turkmenistan Tuvalu Uganda

Ukraine United Arab Emirates United

Kingdom of Great Britain and Northern Ireland

United Republic of Tanzania

United States of America

Uruguay

Uzbekistan

○ Vanuatu ○ Venezuela, Bolivarian Republic of...

○ Viet Nam ○ Yemen ○ Zambia

○ Zimbabwe

Table S3. Marginal means.

Class	Item	Marginal mean	Standard error
COVID-safe mask wearers (class 1)			
	Wears mask if can't socially distance outdoors	1.00	0.001
	Gets tested if cough develops	0.69	0.107
	Goes in public if cough develops	0.11	0.055
	Visits others if cough develops	0.07	-
	Doesn't always use QR codes when entering venues	0.27	0.136
COVID-safe test takers (class 2)			
	Wears mask if can't socially distance outdoors	0.74	0.051
	Gets tested if cough develops	0.94	0.085
	Goes in public if cough develops	<0.01	0.008
	Visit's others if cough develops	<0.01	-
	Doesn't always use QR codes when entering venues	0.28	0.064
COVID-risk isolators (class 3)			
	Wears mask if can't socially distance outdoors	0.29	0.075
	Gets tested if cough develops	0.25	0.108
	Goes in public if cough develops	0.01	-
	Visit's others if cough develops	0.04	-
	Doesn't always use QR codes when entering venues	0.71	0.056
COVID-risk visitors (Class 4)			
	Wears mask if can't socially distance outdoors	0.45	0.044
	Gets tested if cough develops	0.29	0.040
	Goes in public if cough develops	0.91	0.040
	Visit's others if cough develops	0.78	0.041
	Doesn't always use QR codes when entering venues	0.68	0.039

Abbreviations: CI = confidence interval.

Marginal means represent the proportion of the overall sample that responded positively to each item.
